# Obligate heterotrophy of hyperthermophilic archaea *Pyrobaculum arsenaticum* and *P. aerophilum*

**DOI:** 10.1093/femsml/uqaf045

**Published:** 2025-12-27

**Authors:** Eugenio Pettinato, Thomas M Steiner, Christian Seitz, Harald Huber, Wolfgang Eisenreich, Ivan A Berg

**Affiliations:** Institute for Molecular Microbiology and Biotechnology, University of Münster, Münster 48149, Germany; Bavarian NMR Center–Structural Membrane Biochemistry, Department of Chemistry, Technische Universität München, Garching 85747, Germany; Bavarian NMR Center–Structural Membrane Biochemistry, Department of Chemistry, Technische Universität München, Garching 85747, Germany; Institute of Microbiology and Archaea Centre, University of Regensburg, Regensburg 93053, Germany; Bavarian NMR Center–Structural Membrane Biochemistry, Department of Chemistry, Technische Universität München, Garching 85747, Germany; Institute for Molecular Microbiology and Biotechnology, University of Münster, Münster 48149, Germany

**Keywords:** *Pyrobaculum arsenaticum*, *Pyrobaculum aerophilum*, Thermoproteales, heterotrophy, autotrophy, dicarboxylate/4-hydroxybutyrate cycle, 3-hydroxypropionate/4-hydroxybutyrate cycle

## Abstract

*Pyrobaculum arsenaticum* and *P. aerophilum* are two hyperthermophiles that belong to the phylum Thermoproteota (also known as Crenarchaeota), order *Thermoproteales. Pyrobaculum arsenaticum* is an obligate anaerobe, whereas *P. aerophilum* is a facultatively aerobic organism. Both species have been described as capable of autotrophic growth with molecular hydrogen. Because their genomes lack genes encoding key enzymes for known autotrophic CO_2_ fixation pathways, they have been discussed as organisms that may use unknown pathways. To establish reliable autotrophic cultures, we gradually reduced the supplied concentrations of yeast extract but, in our hands, autotrophy was not attainable for either of the two species. Analysis of the ^13^C-labelling of the biomass of the obtained mixotrophic cultures of *P. arsenaticum* grown on ^13^CO_2_ + H_2_ (20:80, v/v), using isotopologue profiling, revealed that their amino acids contained <30% of ^13^C. Amino acids were mainly labelled only in carboxyl groups, demonstrating their purely heterotrophic nature. Our data suggest that the ability to grow autotrophically in currently known *Thermoproteales* is strictly correlated with the presence of the genes for the dicarboxylate/4-hydroxybutyrate cycle. We discuss the reasons that may lead to misinterpretation of the data on the ability of prokaryotes to grow autotrophically.

## Introduction

**Figure 1.**
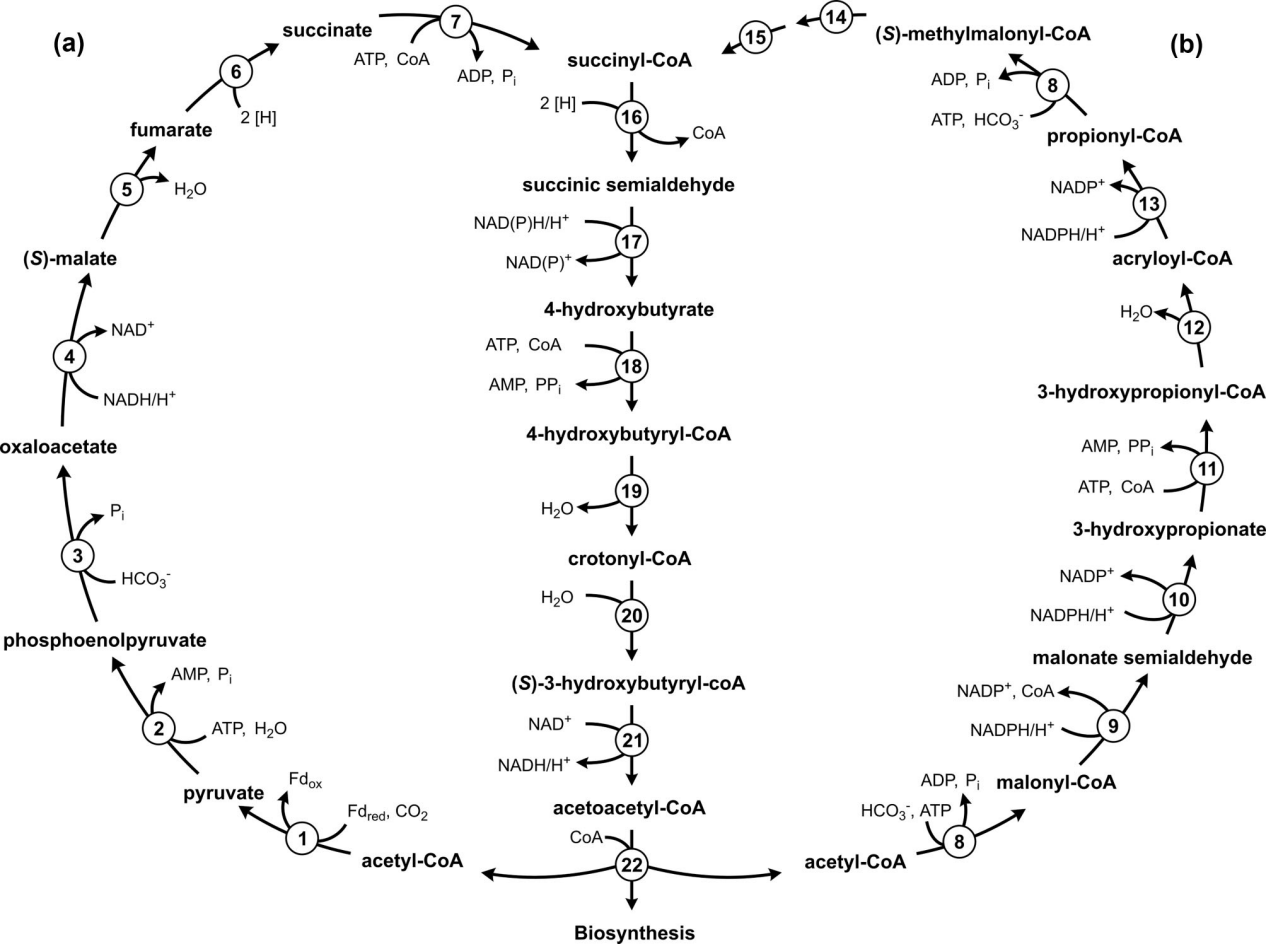
The 4-hydroxybutyrate cycles. (a) The dicarboxylate/4-hydroxybutyrate cycle in *Desulfurococcales* and (b) the 3-hydroxypropionate/4-hydroxybutyrate cycle in *Sulfolobales*. The enzymes are numbered (1) pyruvate synthase, (2) pyruvate:water dikinase, (3) PEP carboxylase, (4) malate dehydrogenase, (5) fumarate hydratase, (6) fumarate reductase (natural electron acceptor is not known), (7) succinyl-CoA synthetase, (8) acetyl-CoA/propionyl-CoA carboxylase, (9) malonyl-CoA reductase, (10) malonic semialdehyde reductase, (11) 3-hydroxypropionate-CoA ligase, (12) 3-hydroxypropionyl-CoA dehydratase, (13) acryloyl-CoA reductase, (14) methylmalonyl-CoA epimerase, (15) methylmalonyl-CoA mutase, (16) succinyl-CoA reductase, (17) succinic semialdehyde reductase, (18) 4-hydroxybutyrate-CoA ligase, (19) 4-hydroxybutyryl-CoA dehydratase, (20) crotonyl-CoA hydratase, (21) (*S*)-3-hydroxybutyryl-CoA dehydrogenase (NAD), and (22) acetoacetyl-CoA ketothiolase. Please note that succinyl-CoA reductase in *Thermoproteales* and *Sulfolobales* is NADPH-dependent, while the electron donor of the enzyme in *Desulfurococcales* is not known.

Of the seven known pathways for inorganic carbon fixation, only three have been described in Archaea (Berg et al. 2010a). The reductive acetyl-CoA (Wood-Ljungdahl) pathway was first discovered in acetogenic bacteria (Ljungdahl 1986, Wood 1991) but was also found to support autotrophy in methanogenic and sulphate-reducing archaea (Euryarchaeota) (Stupperich et al. 1983, Vorholt et al. 1995, 1997, Thauer et al. 2008). In contrast, the 3-hydroxypropionate/4-hydroxybutyrate (HP/HB) and the dicarboxylate/4-hydroxybutyrate (DC/HB) cycles, together referred to as the 4-hydroxybutyrate cycles (Fig. 1), are restricted to archaea and were first discovered in the archaeal phylum Thermoproteota (syn. Crenarchaeota). The HP/HB cycle was elucidated in (micro)aerobic members of the order *Sulfolobales* (*Metallosphaera sedula*) (Berg et al. 2007). A convergently evolved variant of this pathway is also responsible for autotrophic CO_2_ fixation in ammonia-oxidizing archaea (Könneke et al. 2014, Otte et al. 2015). The DC/HB cycle functions in anaerobic *Desulfurococcales* (*Ignicoccus hospitalis, Pyrolobus fumarii*) (Berg et al. 2010a, Jahn et al. 2007, Huber et al. 2008, 2010b, Flechsler et al. 2021) and *Thermoproteales* (*Pyrobaculum neutrophilum*, formerly *Thermoproteus neutrophilus*) (Ramos-Vera et al. 2009).

The HP/HB and DC/HB cycles allow the fixation of two inorganic carbons with acetyl-CoA as the primary acceptor molecule (Fig. 1). The direct fixation product of both cycles is succinyl-CoA, nonetheless distinct carboxylating enzymes are used. The HP/HB cycle utilizes the characteristic biotin-dependent acetyl-CoA/propionyl-CoA carboxylases (Hügler et al. 2003, Berg et al. 2007), whereas the DC/HB cycle uses pyruvate synthase and phosphoenolpyruvate (PEP) carboxylase (Jahn et al. 2007, Huber et al. 2008). The use of different enzymes and electron donors determines the difference in oxygen tolerance of these two cycles. The DC/HB cycle functions in anaerobic archaea due to oxygen sensitivity of ferredoxin-dependent pyruvate synthase. Furthermore, it was hypothesized that some other enzymes of the cycle (e.g. fumarate reductase and succinyl-CoA reductase in *I. hospitalis* and *P. fumarii*) may utilize ferredoxin as electron donor (Huber et al. 2008, Berg et al. 2010). In contrast, the enzymes of the HP/HB cycle are oxygen tolerant (Hügler et al. 2003, Berg et al. 2007, Alber et al. 2008, Teufel et al. 2009, Kockelkorn and Fuchs 2009, Han et al. 2012, Hawkins et al. 2013, 2014, Könneke et al. 2014, Liu et al. 2020, 2021a, 2021b).

The regeneration of acetyl-CoA from succinyl-CoA is similar in both pathways (Fig. 1). Succinyl-CoA is reduced to 4-hydroxybutyrate, which is activated to 4-hydroxybutyryl-CoA and then dehydrated to crotonyl-CoA by 4-hydroxybutyryl-CoA dehydratase. The latter enzyme is a [4Fe–4S] and FAD-containing dehydratase (Martins et al. 2004, Buckel and Golding 2006) that is considered to be the key enzyme of both cycles. Its product, crotonyl-CoA, is further converted to acetoacetyl-CoA and then to two acetyl-CoA molecules, closing the cycle and generating one additional molecule of acetyl-CoA for biosynthesis.

The differences between the two cycles correlate with the conditions under which the corresponding archaea are able to grow. Nevertheless, the order *Sulfolobales* includes, besides aerobic or microaerobic representatives, obligately anaerobic organisms (e.g. *Stygiolobus azoricus*, Segerer et al. 1991). Similarly, members of the *Desulfurococcales*, being generally anaerobic, contain facultatively aerobic autotrophic representatives as well [e.g. *Pyrolobus fumarii* (Blöchl et al. 1997)]. It was shown that the distribution of these cycles correlates with archaeal phylogeny rather than with the aerobic or anaerobic lifestyle of the organisms in question (Berg et al. 2010b). Thus, to date, the functioning of the HP/HB or DC/HB cycle in uncharacterized microorganisms could be predicted based on phylogenetic affiliations (i.e. *Sulfolobales, Desulfurococcales*, or *Thermoproteales*) and/or genomic analysis (i.e. presence of genes encoding key enzymes, such as 4-hydroxybutyryl-CoA dehydratase). However, ambiguous cases among the sequenced *Thermoproteales* have to be studied experimentally.

Today, 14 species of the order *Thermoproteales* have been fully sequenced, and their genomes are available. Table 1 shows the distribution of homologues of the genes from the DC/HB cycle gene cluster of *P. neutrophilum* (Ramos-Vera et al. 2009). Organisms that were described as capable of autotrophy (i.e. *Pyrobaculum islandicum, Thermoproteus tenax*) as well as some apparently obligately heterotrophic representatives (i.e. *Pyrobaculum ferrireducens, Pyrobaculum calidifontis, Pyrobaculum yellowstonensis*) harbor the DC/HB cycle gene cluster in their genomes (Table 1). The opposite case has also been shown, as some *Thermoproteales* isolated as autotrophs do not possess some of the genes of the *P. neutrophilum* cluster.

**Table 1.** Gene homologues involved in autotrophic CO_2_ assimilation via the DC/HB cycle present in sequenced *Thermoproteales* compared with *P. neutrophilum*.

Protein queries and accession from *P. neutrophilum*	*Pyrobaculum arsenaticum* PZ6 DSM 13 514	*Pyrobaculum aerophilum* IM2	*Pyrobaculum oguniense* TE7 DSM 13 380	*Pyrobaculum islandicum* DSM 4184	*Pyrobaculum yellowst-onensis WP30*	*Pyrobaculum ferrireducens* 1860	*Pyrobaculum calidifontis* JCM 11 548	*Thermop-roteus tenax* Kra1	*Thermop-roteus uzoniensis* 768–20	*Vulcan-isaeta moutnovskia* 768–28	*Vulcani-saeta distributa* DSM 14 429	*Vulcanisaeta souniana JCM 11 219*	*Caldivirga maquil-ingensis* IC-167
**PEP carboxylase (ACB39366.1)**	ABP50594.1 (64/78)	AAL64899.1 (53/67)	(AFA39345.1, 64/78)	ABL87432.1 (84/95)	AKT34101.1 (62/82)	AET32163.1 (69/84)	ABO08812.1 (65/78)	CCC81750.1 (66/83)	AEA12427.1 (65/82) (AEA13124.1, 50/67)	**Absent** (NA)	**Absent** (ADN50074.1,31/46)	**Absent** (BDR92534.1,30/48)	**Absent** (ABW02733.1, 30/46)
**Succinic semialdehyde reductase (ACB39367.1)**	**Absent** (ABP49998.1.39/57)	**Absent** (AAL64544.1,38/57)	**Absent** (AAL64544.1,38/57)	ABL87431.1 (97/98)	AKT34100.1 (77/82)	AET32164.1 (87/89)	ABO08811.1 (74/88)	CCC81749.1 (72/85)	AEA12428.1 (72/86)	**Absent** (ADY01576.1,38/55)	**Absent** (ADN49671.1(39/55)	**Absent** (BDR92843.1, 45/61)	**Absent** (ABW02542.1,39/53)
**putative 4HB-CoA-ligase (ACB39368.1)**	**Absent** (ABP50528.1,49/66)	**?** AAL64220.1 (54/72)	AFA39323.1 (80/89)	ABL87430.1 (98/99) (ABL87449.1,50/67)	AKT34094.1 (81/91)	AET32171.1 (83/91)	ABO08806.1 (81/90) (ABO07815.1, 50/66)	CCC81743.1 (82/89)	(AEA12398.1, 82/90)	**?** (ADY00302.1, 50/66)	**?** (ADN50914.1, 50/97)	**Absent** (BDR91637.1, 48/65)	**Absent** (ABW01104.1,47/64)
**Succinyl-CoA reductase (ACB39369.1)**	**Absent** (ABP50149.1,34/53)	**Absent** (AAL64225.1,38/57)	AFA39324.1 (72/86)	ABL87429.1 (96/98)	AKT34095.1 (73/85)	AET32169.1 (75/84)	ABO08807.1 (73/85)	CCC81744.1 (72/85)	AEA12434.1 (71/85)	(ADY01372.1, 36/57)	**Absent** (ADN51031.1,37/57)	**Absent** (BDR91798.1, 38/57)	**Absent** (ABW00927.1,42/62)
**4HB-CoA-dehydratase (ACB39370.1)**	**Absent** (NA)	AAL64371.1 (75/88)	AFA39325.1 (74/86)	ABL87428.1 (95/98)	AKT34096.1 (80/91)	AET32168.1 (77/89)	(ABO08816.1, 78/88)	CCC81745.1 (77/89)	AEA12432.1 (74/87)	**Absent** (ADY00892.1,33/54)	**Absent** (ADN51582.1,35/54)	(BDR92517.1, 78/89)	**Absent** (NA)
**putative fumarate reductase SdrA/FdrA (ACB39371.1)**	ABP51901.1 (58/73)	AAL62973.1 (59/73)	AFA40534.1 (58/74)	ABL87427.1 (91/94) (ABL87849.1, 59/73)	AKT34097.1 (74/84) (AKT35871.1, 61/74)	AET32167.1 (74/85) (AET33606.1, 59/73)	ABO08808.1 (68/81) (ABO09542.1, 60/74)	CCC81747.1 (63/76) (CCC81519.1, 59/74)	AEA12430.1 (64/77) (AEA12099.1, 58/73)	(ADY00468.1, 52/68)	(ADN51185.1, 52/69)	(BDR91117.1, 53/69)	(ABW02686.1, 51/68)
**putative fumarate reductase SdrB/FdrB (ACB39372.1)**	(ABP51902.1, 49/70)	(AAL62974.1, 49/68) (AAL64369.1, 56/71)	AFA39327.1 (55/77) (AFA40533.1, 47/70)	ABL87426.1 (85/91) (ABL87848.1, 57/72)	AKT34098.1 (71/82) (AKT35872.1, 53/71)	AET32166.1 (70/81) (AET33607.1, 49/68)	ABO08809.1 (55/74)	CCC81748.1 (63/79)	AEA12429.1 (57/74) (AEA12100.1, 49/69)	(ADY01533.1, 50/64)	(ADN49711.1, 50/65)	(BDR92548.1, 52/65)	(ABW02181.1, 57/70)
**putative nucleotide-disulfide oxidoreductase (ACB39373.1)**	(ABP51126.1, 49/64)	(AAL64145.1, 48/63)	AFA39328.1 (52/66)	ABL87425.1 (95/97)	AKT34099.1 (62/73) (AKT35219.1, 48/61)	AET32165.1 (66/78) (AET31951.1, 49/64)	ABO08810.1 (54/68) (ABO09117.1, 50/64)	(CCC82057.1, 49/63)	(AEA13586.1. 49/64)	(ADY01126.1, 43/60)	(ADN49444.1, 44/61)	(BDR90938.1, 43/61)	(ABW01951.1, 43/59)
**Nutrition and references**	Autotrophic (Huber et al. 2000)	Autotrophic (Völkl et al. 1993)	Heterotrophic (Sako et al. 2001)	Autotrophic (Hu and Holden 2006)	Heterotrophic (Jay et al. 2015)	Heterotrophic (Slobodkina et al. 2015)	Heterotrophic (Amo et al. 2002)	Autotrophic (Zillig et al. 1981, Fischer et al. 1983)	Autotrophic (Mardanov et al. 2011)	Heterotrophic (Gumerov et al. 2011)	Heterotrophic (Itoh et al. 2002)	Heterotrophic (Itoh et al. 2002)	Heterotrophic (Itoh et al. 1999)

Note that these genes are co-localized in the genomes of *P. neutrophilum* and other (potentially) autotrophic *Thermoproteales*. The amino acid sequence identity/similarity to *P. neutrophilum* enzymes (in %) is shown in parentheses. Genes in parentheses were excluded from Fig. 4, while underlined genes were included. Although the homologues of PEP carboxylase, succinic semialdehyde reductase, 4-hydroxybutyrate-CoA ligase, succinyl-CoA reductase, and 4-hydroxybutyryl-CoA dehydratase with sequence identity >30%, if present, are shown in the table, the homologues of other proteins with sequence identity <50% are regarded as having another function (marked as ‘absent’).

Homologues with >70% query coverage and >30% protein identity were included, results not matching these parameters were excluded and the gene was reported as NA (not applicable).

*Pyrobaculum aerophilum* (GenBank: AE009441.1) and *P. arsenaticum* (GenBank: CP000660.1) were described as capable of autotrophy with molecular hydrogen but lack many genes for the enzymes catalyzing specific reactions of the 4-hydroxybutyrate part of the cycle (Table 1) (Völkl et al. 1993, Huber et al. 2000, Fitz-Gibbon et al. 2002). Although *P. aerophilum* possesses genes encoding 4-hydroxybutyryl-CoA dehydratase and a putative 4-hydroxybutyrate-CoA ligase, other specific enzymes of the pathway are absent in its genome (i.e. succinic semialdehyde reductase and succinyl-CoA reductase). Furthermore, *P. arsenaticum* does not possess genes for any specific enzyme of the DC/HB cycle (Table 1) or any other known carbon dioxide fixation pathways. Therefore, it is unclear whether these species grow by means of a modified DC/HB cycle with the corresponding reactions catalyzed by nonhomologous enzymes, or operate a still undiscovered CO_2_ fixation pathway, or were mistakenly assigned to autotrophs. Accordingly, we started a more detailed investigation of their autotrophy.

## Materials and methods

### Chemicals

Chemicals, biochemicals and Neubauer counting chambers were obtained from Sigma-Aldrich, Merck, Roth, or VWR.

### Microbial strains and growth conditions

*Pyrobaculum arsenaticum* (DSM 13514) and *P. aerophilum* (DSM 7523) were obtained from the culture collection of the German Archaea Centre (University of Regensburg, Lehrstuhl für Mikrobiologie & Archaeenzentrum, Dr. H. Huber). *Pyrobaculum arsenaticum* was cultivated in minimal 1/20 MG-CB medium (Huber et al. 2000) containing 0.9 g l^−1^ NaCl, 0.215 g l^−1^ MgCl_2_·6H_2_O, 0.017 g l^−1^ KCl, 0.007 g l^−1^ K_2_HPO_4_·3H_2_O, 0.012 g l^−1^ NH_4_Cl, 0.007 g l^−1^ CaCl_2_·2H_2_O, 0.05 g l^−1^ CaCO_3_, 0.1 mg l^−1^ (NH_4_)_2_ Fe(SO_4_)_2_·6H_2_O, 1 ml l^−1^ of mineral solution, and 1 ml l^−1^ Wolfe’s vitamin solution. The mineral solution contained 580 mg l^−1^ MnCl_2_ x 4H_2_O, 1 g l^−1^ NaCl, 700 mg l^−1^ FeCl_2_⋅4H_2_O, 180 mg l^−1^ CoCl_2_⋅6H_2_O, 100 mg l^−1^ CaCl_2_⋅2H_2_O, 84 mg l^−1^ ZnCl_2_, 7 mg l^−1^ CuCl_2_⋅2H_2_O, 10 mg l^−1^ H_3_BO_3_, 5 mg AlCl_3_, 10 mg l^−1^ Na_2_MoO_4_⋅2H_2_O, 48 mg l^−1^ NiCl⋅6H_2_O, 10 mg l^−1^ Na_2_WO_4_⋅2H_2_O, and 10 mg l^−1^ Na_2_SeO_4_. The vitamin solution contained 20 mg l^−1^ biotin, 20 mg l^−1^ folic acid, 100 mg l^−1^ pyridoxamine dihydrochloride, 50 mg l^−1^ thiamine dihydrochloride, 50 mg l^−1^ riboflavin, 50 mg l^−1^ nicotinic acid, 50 mg l^−1^ DL-Ca-pantothenate, 1 mg l^−1^ cyanocobalamin, 50 mg l^−1^ 4-aminobenzoic acid, and 50 mg l^−1^ lipoic acid.

*Pyrobaculum aerophilum* was cultivated in minimal BS medium (Völkl et al. 1993) containing 0.25 g l^−1^ NH_4_Cl, 0.07 g l^−1^ K_2_HPO_4_, 125 ml l^−1^ of synthetic seawater (marine) medium, and 1 ml l^−1^ of mineral solution. The synthetic marine medium contained 47.15 g l^−1^ NaCl, 7 g l^−1^ MgSO_4_⋅7H_2_O, 18.1 g l^−1^ MgCl_2_⋅6H_2_O, 3.13 g l^−1^ CaCl_2_ x 2 H_2_O, 3.24 g l^−1^ Na_2_SO_4_, 1.20 g l^−1^ KCl, 195 mg l^−1^ KBr, 72 mg l^−1^ SrCl_2_⋅6H_2_O, 52 mg l^−1^ H_3_BO_3_, 2.4 mg l^−1^ NaF, 400 µg l^−1^ Na_2_SO_3_⋅5H_2_O, and 50 µg l^−1^ KI. The mineral solution contained 30 g l^−1^ MgSO_4_⋅7 H_2_O, 5 g l^−1^ MnSO_4_⋅H_2_O, 10 g l^−1^ NaCl, 1 g l^−1^ FeSO_4_⋅7H_2_O, 1.8 g l^−1^ CoSO_4_⋅7H_2_O, 1 g l^−1^ CaCl_2_⋅2H_2_O, 1.8 g l^−1^ ZnSO_4_⋅7H_2_O, 100 mg l^−1^ CuSO_4_⋅5H_2_O, 180 mg l^−1^ KAl (SO_4_)_2_⋅12H_2_O, 100 mg l^−1^ H_3_BO_3_, 100 mg l^−1^ Na_2_MoO_4_⋅2H_2_O, 2.8 g l^−1^ (NH_4_)_2_Ni(SO_4_)_2_⋅6H_2_O, 100 mg l^−1^ Na_2_WO_4_⋅2H_2_O, and 100 mg l^−1^ Na_2_SeO_4._

Both media were prepared without supplements, namely electron acceptors (i.e. arsenate or nitrate), vitamin solution, and bicarbonate, made anaerobic by bubbling with N_2_ (100%) and dispensed anaerobically into serum bottles in an anaerobic chamber. The bottles were sealed with butyl rubber stoppers and aluminium caps and autoclaved for 20 min at 121°C. Before inoculation, the medium was reduced by the addition of Na_2_S·9H_2_O to a final concentration of 0.05% (w/v) and supplemented with vitamins and electron acceptor. Arsenate was supplemented to *P. arsenaticum* cultures at a final concentration of 0.2% (w/v), whereas nitrate was supplemented to *P. aerophilum* cultures at a final concentration of 0.1% (w/v). Before inoculation, during the test phase in which we tried gradually reducing yeast extract supplement, we added it to a final concentration of 0.1%, 0.01%, or 0.001% w/v. The gas phase was replaced with H_2_:CO_2_ (80:20, v/v) at two bars. Sodium bicarbonate solution (8%, w/v) was used to adjust the pH to an optimum of 7.0 for both organisms, at an approximate final concentration of bicarbonate of 0.2% (w/v). Cultures were incubated at 95°C.

### Preparation of samples for mass-spectrometry analysis

Isolation of protein bound amino acids was done as described previously (Eylert et al. 2008). About 2 mg of bacterial sample (lyophilized cell pellet) was suspended in 500 μl of 6 M HCl and hydrolyzed overnight at 105°C. The reaction mixture was dried under a stream of nitrogen. The residue was suspended in 200 μl of 50% acetic acid. Amino acids were isolated using a small column of Dowex 50 W X8 (7 × 10 mm; 200–400 mesh, 34–74 μm, H^+^-form). The column was first washed with 2 ml H_2_O, then amino acids were eluted with 1 ml 4 M aqueous ammonia solution. The ammonia eluate was dried under a stream of nitrogen at 70°C.

Acid hydrolysis leads to the conversion of glutamine and asparagine to glutamate and aspartate, respectively. Therefore, results given for aspartate and glutamate correspond to asparagine or aspartate and glutamine or glutamate, respectively. To account for different derivatisation and ionization efficiency of each amino acid, an equimolar amino acid mixture (2.5 μM in 0.1 M HCl) was used to determine the response factor for each amino acid. Therefore, 200 μl of the amino acid mixture was dried under a stream of nitrogen at 70°C. The dried residue was treated with 50 μl *N*-(*tert*-butyldimethylsilyl)-*N*-methyltrifluoroacetamide (MTBSTFA) containing 1% *N*-*tert*-butyldimethylsilylchloride (TBDMS) and 50 μl anhydrous acetonitrile at 70°C for 30 min.

For analysis of glycerol content, 1 ml of *P. arsenaticum* 1/20 MG-CB medium, vitamin stock, or arsenate stock solutions were dried under N_2_ flux. The residue was suspended in 1 mL of cold methanol (HPLC-grade, 4°C) and was centrifuged (10 000 × *g* for 10 min, 4°C). 5 µl of a 10 mM norvaline solution (in methanol, internal standard) was added to the supernatant. The mixture was then dried under N_2_ flux. As for the amino acids, the residue was treated with 50 µl of MTBSTFA containing 1% TBDMS and 50 µl of water-free acetonitrile at 70°C for 1 h. The TBDMS derivatives were then analysed by gas chromatography–mass spectrometry (GC–MS).

### GC–MS analysis

GC–MS analysis was performed with a QP2010 Plus GC–MS (Shimadzu) equipped with a fused silica capillary column (Equity TM-5; 30 m × 0.25 mm, 0.25-μm film thickness; SUPELCO) and a quadrupole detector working with electron impact ionization at 70 eV. An aliquot (0.1–6 μl) of the TBDMS-derivatised samples was injected in 1:5 split mode at an interface temperature of 260°C and a helium inlet pressure of 70 kPa.

For the analysis of ^13^C excess and isotopologue composition of bacterial amino acids, selected ion monitoring was used with a sampling rate of 0.5 s and LABSOLUTION software (Shimadzu) was used for data collection and analysis. Isotopologue calculations were performed for m/z [M–57]^+^ or m/z [M–85]^+^. For analysis of the relative amino acid composition in *P. arsenaticum* protein as well as the medium composition, measurements were performed in scan mode in a mass range from 45 m/z to 700 m/z with an injection volume of 0.1 μl.

For amino acids, the column was heated to 150°C and kept at 150°C for 3 min, after which was heated to 280°C (7°C per min) and held at that temperature for 3 min. Measurements were performed in SCAN mode with a scan interval of 0.5 s and a mass range of 50–600 m/z. All experiments were performed in three biological replicates, which were measured three times for technical replicates. The calculation of ^13^C excess was done as described previously (Eylert et al. 2008) and comprises (i) the detection of GC–MS spectra of unlabelled derivatized metabolites; (ii) the determination of the absolute mass of isotopologue enrichments and distributions of labelled metabolites of the experiment; and (iii) the correction of the absolute ^13^C incorporation by subtracting the contributions of the heavy isotopologues due to the natural abundances in the derivatized metabolites to calculate the enrichments and distributions of the isotopologues. For the ^13^C-labelling data analysis, Isotopo-4 software was used (Ahmed et al. 2014).

The analysis of the silylated glycerol products was performed with GC–MS using the same equipment as described above and an AOC-20i auto injector. Temperature program and settings were the following: 0–6 min at 60°C; 6–25 min at 60°C–280°C, 12°C/min; 25–28 min at 280°C; injector temperature: 260°C; detector temperature: 260°C; column flow rate: 1 ml/min; scan interval: 0.5 s; and injection volume 0.2 μl. Retention time of acetate: 6.69 min; retention time of glycerol: 19.94 min.

## Results and discussion

Similar to other members of the order *Thermoproteales, P. aerophilum*, and *P. arsenaticum* are hyperthermophilic organisms growing at an optimum temperature of approximately 95°C–100°C. *P. aerophilum* was described as being capable of chemolithoautotrophic growth with hydrogen or thiosuphate as electron donor, performing aerobic respiration or dissimilatory nitrate reduction (Völkl et al. 1993). *Pyrobaculum arsenaticum* was the first reported organism with the ability to grow anaerobically by chemolithoautotrophic reduction of arsenate or selenate, potentially contributing to the biogeochemical cycling of heavy metals in high-temperature environments (Huber et al. 2000). In our laboratory, both strains were grown under mixotrophic conditions at 95°C with mineral medium supplemented with yeast extract and with H_2_:CO_2_ (80:20, v/v) as the gas phase. Arsenate for *P. arsenaticum* and nitrate for *P. aerophilum* were provided as electron acceptors. In the present study, stable cultures reliably growing autotrophically were not achieved for either strain.

### Analysis of *P. arsenaticum* autotrophy

*Pyrobaculum arsenaticum* first appeared to grow autotrophically. After four consecutive re-inoculations without yeast extract supplementation to the minimal medium, a growth from 10^5^ up to 10^7^ cells/ml could be detected. Hence, we performed a growth experiment with ^13^CO_2_ and molecular hydrogen (H_2_:^13^CO_2_, 80:20, v/v) to give a deeper look in the actual metabolism of this species. Although the culture replicates were treated identically and incubated under the same conditions (95°C; 100 rpm), the cultures grew at different final cell densities (from no growth to 2.7 × 10^7^ cells/ml) and did not show comparable growth behaviour. Nevertheless, the biomass obtained from the best performing cultures was used to perform isotopologue profiling analysis of the amino acids.

**Figure 2.**
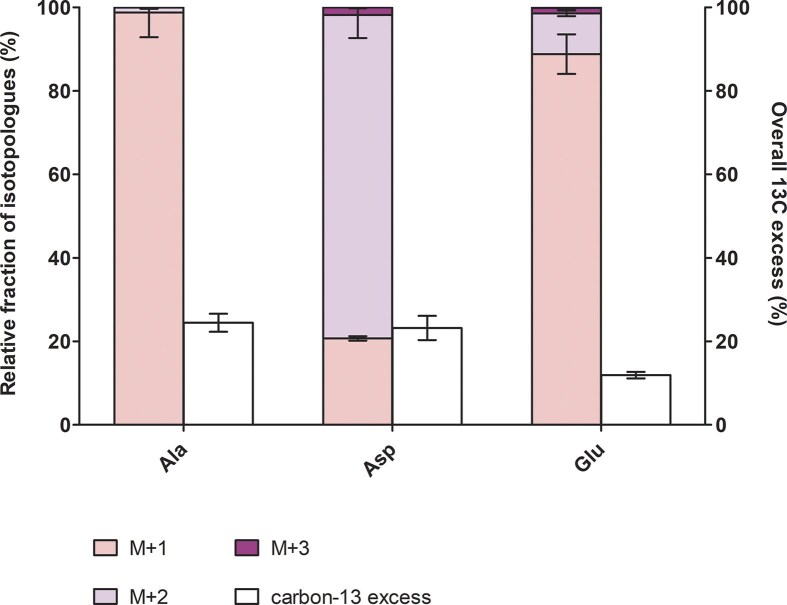
Isotopologue profiling of the amino acids of *P. arsenaticum* supposedly autotrophic cultures. ^13^C-isotopologue fractions (normalized to 100%) and ^13^C excess after apparent autotrophic growth with H_2_, arsenate (HAsO_4_^2-^), and 20% ^13^CO_2_. Data are mean ± s.e.m. of three biological replicates.

The isotopologue profiling patterns showed that the amino acids fragments obtained from the collected biomass of supposedly autotrophic *P. arsenaticum* cultures were mainly unlabelled or M+1 and M+2 labelled (Fig. 2, Table 2), with ‘*M*’ indicating the mass of the parental fragment plus the number of carbon-13 present in the molecule. No fully labelled amino acids were detected, indicating the heterotrophic nature of the growth of this archaeon. This is in sharp contrast to the results obtained in our study of CO_2_ fixation in *Hippea maritima*, which grows on H_2_/CO_2_ gas mixtures using the reversed oxidative TCA cycle, but only in the presence of yeast extract, i.e. strictly mixotrophically (Steffens et al. 2021, 2022). Although this bacterium only built up to 16% of the carbon of its amino acids from ^13^CO_2_, a significant amount of fully labelled amino acids was detected (e.g. 14.4% of M+4 aspartate and 22.3% of M+5 glutamate), indicating the operation of a closed autotrophic pathway (Steffens et al. 2021, 2022).

**Table 2.** ^13^C-Enrichment in protein derived amino acids from *P. arsenaticum* grown with ^13^CO_2_/H_2_ analysed as tert-butyl-dimethylsilyl derivatives. ND, not detected.

Aminoacid	Isotopologues	Replicate 1		Replicate 2		Replicate 3		Combined	
		Average	St. dev.	Average	St. dev.	Average	St. dev.	Average	St. dev.
Ala	M+1	74.78%	0.16%	64.74%	0.38%	75.18%	0.25%	71.57%	5.92%
	M+2	1.11%	0.07%	0.58%	0.07%	0.92%	0.13%	0.87%	0.28%
	M+3	0.01%	0.02%	0.01%	0.02%	0.00%	0.01%	0.01%	0.02%
Asp	M+1	10.71%	0.12%	10.10%	0.32%	11.04%	0.16%	10.62%	0.52%
	M+2	37.25%	0.65%	35.88%	1.00%	46.01%	0.52%	39.71%	5.55%
	M+3	0.81%	0.11%	0.98%	0.29%	0.97%	0.24%	0.92%	0.23%
	M+4	0.00%	0.00%	0.00%	0.00%	0.00%	0.00%	0.00%	0.00%
Glu	M+1	45.54%	0.29%	43.06%	2.17%	51.99%	0.83%	46.86%	4.74%
	M+2	5.54%	0.18%	5.18%	1.25%	4.79%	0.29%	5.17%	0.68%
	M+3	0.75%	0.09%	0.68%	0.61%	0.60%	0.10%	0.68%	0.28%
	M+4	0.05%	0.06%	0.02%	0.03%	0.05%	0.04%	0.04%	0.05%
	M+5	0.02%	0.02%	0.05%	0.07%	0.00%	0.00%	0.02%	0.04%
Gly	M+1	52.88%	0.12%	36.10%	0.62%	51.61%	0.23%	46.86%	9.35%
	M+2	0.19%	0.08%	0.05%	0.09%	0.10%	0.09%	0.11%	0.11%
Ile	M+1	20.94%	0.27%	24.12%	0.31%	29.25%	0.46%	24.77%	4.21%
	M+2	0.47%	0.18%	0.27%	0.13%	0.25%	0.20%	0.33%	0.21%
	M+3	0.45%	0.07%	0.49%	0.01%	0.61%	0.10%	0.52%	0.11%
	M+4	0.00%	0.00%	0.06%	0.04%	0.02%	0.02%	0.03%	0.04%
	M+5	0.09%	0.03%	0.58%	0.02%	0.15%	0.06%	0.27%	0.27%
Leu	M+1	1.66%	0.36%	1.43%	0.22%	2.15%	0.06%	1.75%	0.42%
	M+2	0.01%	0.02%	0.04%	0.05%	0.09%	0.05%	0.05%	0.05%
	M+3	0.03%	0.00%	0.00%	0.00%	0.03%	0.03%	0.02%	0.02%
	M+4	0.00%	0.00%	0.00%	0.00%	0.00%	0.00%	0.00%	0.00%
	M+5	0.05%	0.01%	0.15%	0.02%	0.06%	0.01%	0.08%	0.06%
Phe	M+1	3.80%	0.11%	3.91%	0.34%	3.39%	0.21%	3.70%	0.35%
	M+2	17.36%	0.17%	13.77%	0.54%	15.49%	0.44%	15.54%	1.84%
	M+3	51.60%	0.32%	41.03%	0.36%	52.48%	0.38%	48.37%	6.38%
	M+4	3.22%	0.14%	2.57%	0.19%	3.29%	0.18%	3.03%	0.43%
	M+5	0.46%	0.17%	0.88%	0.13%	0.81%	0.13%	0.72%	0.26%
	M+6	0.23%	0.07%	0.62%	0.11%	0.48%	0.13%	0.44%	0.23%
	M+7	0.12%	0.02%	0.12%	0.06%	0.09%	0.07%	0.11%	0.05%
	M+8	0.00%	0.00%	0.13%	0.18%	0.01%	0.02%	0.05%	0.10%
	M+9	0.00%	0.00%	0.00%	0.00%	0.00%	0.00%	0.00%	0.00%
Pro	M+1	53.07%	0.30%	63.29%	1.26%	61.50%	0.73%	59.29%	5.51%
	M+2	2.50%	0.13%	1.00%	0.17%	2.00%	0.21%	1.83%	0.78%
	M+3	0.09%	0.07%	0.00%	0.00%	0.00%	0.00%	0.03%	0.06%
	M+4	0.25%	0.01%	0.19%	0.12%	0.20%	0.08%	0.22%	0.08%
	M+5	0.08%	0.02%	0.06%	0.01%	0.11%	0.04%	0.08%	0.04%
Ser	M+1	58.76%	0.46%	44.92%	0.59%	58.58%	0.61%	54.09%	7.96%
	M+2	3.19%	0.73%	1.47%	0.37%	2.26%	0.72%	2.31%	1.05%
	M+3	0.03%	0.05%	0.25%	0.22%	0.03%	0.05%	0.10%	0.17%
Thr	M+1	11.62%	0.53%	10.93%	0.37%	11.54%	0.22%	11.36%	0.53%
	M+2	36.19%	0.23%	33.91%	0.14%	44.65%	0.63%	38.25%	5.67%
	M+3	1.50%	0.23%	1.28%	0.12%	1.90%	0.08%	1.56%	0.34%
	M+4	0.00%	0.00%	0.00%	0.00%	0.01%	0.02%	0.00%	0.01%
Tyr	M+1	4.97%	2.44%	2.91%	2.15%	ND	ND	3.94%	2.72%
	M+2	20.45%	1.41%	15.32%	3.75%	ND	ND	17.89%	4.45%
	M+3	55.83%	7.60%	59.42%	5.11%	ND	ND	57.63%	6.84%
	M+4	6.26%	1.32%	4.28%	2.55%	ND	ND	5.27%	2.39%
	M+5	2.71%	3.81%	0.76%	1.07%	ND	ND	1.73%	2.80%
	M+6	0.26%	0.44%	0.13%	0.23%	ND	ND	0.19%	0.35%
	M+7	0.09%	0.15%	0.34%	0.59%	ND	ND	0.21%	0.41%
	M+8	0.31%	0.30%	1.49%	1.54%	ND	ND	0.90%	1.24%
	M+9	1.92%	3.26%	2.47%	4.04%	ND	ND	2.19%	3.67%
Val	M+1	77.06%	0.15%	69.18%	0.13%	76.08%	0.64%	74.11%	4.30%
	M+2	1.53%	0.07%	1.28%	0.35%	1.52%	0.21%	1.45%	0.25%
	M+3	0.06%	0.03%	0.06%	0.11%	0.03%	0.03%	0.05%	0.06%
	M+4	0.00%	0.00%	0.01%	0.01%	0.00%	0.00%	0.00%	0.01%
	M+5	0.00%	0.00%	0.03%	0.03%	0.00%	0.01%	0.01%	0.02%

In order to determine the source of the organic carbon contamination explaining the detected weak growth, we analysed all solutions used for the medium preparation via GC-MS analysis. Indeed, the presence of glycerol was detected in fresh noninoculated medium, in medium after inoculation, and in the stock solution of vitamins (i.e. 215 µM, 258 µM, and 313 µM, respectively). As vitamin solutions were sterilized by filtration using cellulose acetate syringe filters (0.2 µm pore size, VWR, catalogue number: 514-0061), we assumed that the glycerol contamination was caused by the filters, since glycerol was not detected in unfiltered medium (<0.2 µM), and since commercial filters are often stored with a glycerol coverage. This amount of glycerol (200 µM = ~18 mg/l) is sufficient to explain the observed growth, given that the cells grew in the presence of molecular hydrogen. Indeed, 10^7^ cells/ml corresponds to ~5 mg/l of dry weight, as the dry mass of a *Pyrobaculum* cell is comparable to that of an *Escherichia coli* cell (0.5 pg, Phillips and Milo 2009).

Although the ability of *Pyrobaculum* spp. to utilize glycerol as a sole carbon source has not been described until now (Völkl et al. 1993, Huber et al. 2000), it is not unlikely. Among Archaea, the ability to use glycerol as a carbon and energy source is widespread among haloarchaea (Williams et al. 2017), and has recently been demonstrated in *Sulfolobus acidocaldarius* (Schmerling et al. 2025) though it has not yet been studied in hyperthermophiles. Glycerol (i.e. *sn*-glycerol-1-phosphate) forms the backbone of the archaeal monolayer membrane lipids. Archaeal membranes are mainly composed of two types of isoprenoid ether lipids and possess different features compared to bacterial or eukaryotic membranes, conferring competitive advantage in extreme environments (Albers and Meyer 2011). For example, dialkylglycerol tetraethers with varying degrees of cyclization were detected as core lipids in cell membrane of *P. aerophilum* (Völkl et al. 1993).

Microbial glycerol metabolism proceeds via dihydroxyacetone phosphate, which is formed either via glycerol kinase and glycerol-3-phosphate dehydrogenase, or through glycerol dehydrogenase and dihydroxyacetone kinase (Bräsen et al. 2014, Klein et al. 2017, Williams et al. 2017). While haloarchaea use both these pathways (Nishihara et al. 1999, Falb et al. 2008, Sherwood et al. 2009, Williams et al. 2017), *S. acidocaldarius* catabolizes glycerol through glycerol-3-phosphate dehydrogenase reaction (Schmerling et al. 2025). However, the genome of *P. arsenaticum* does not encode the genes for the key enzymes of these pathways (glycerol kinase, glycerol-3-phosphate dehydrogenase, dihydroxyacetone kinase and glycerol dehydrogenase), but it does encode a gene annotated as *sn*-glycerol-1-dehydrogenase (ABP51378), which shares 44/60% and 41/58% amino acid sequence identity/similarity, respectively, with *sn*-glycerol-1-dehydrogenases (BAA79484; BAA13644) from the aerobic hyperthermophilic archaeon, *Aeropyrum pernix* K1 (Han et al. 2002), and from the anaerobic thermophilic methanogenic archaeon *Methanothermobacter thermautotrophicus* (Nishihara and Koga 1997). Glycerol utilization may therefore proceed via nonhomologous proteins, or glycerol could be phosphorylated to glycerol-1-phosphate via a novel kinase. Either way, glycerol metabolism in *Pyrobaculum* spp. requires further experimental investigation.

The labelling patterns obtained are most likely the result of various carboxylation reactions: M+1 labelling in alanine is probably the result of an exchange reaction of the carboxylic group of pyruvate with the ^13^CO_2_ in the medium, which is catalyzed by pyruvate synthase in addition to pyruvate conversion to acetyl-CoA (Raeburn and Rabinowitz 1971). M+1 or M+2 in aspartate are the result of pyruvate (or PEP) carboxylation. Glutamate was predominantly M+1 labelled (46.9 ± 4.7%) with traces of M+2 and M+3 labelling (5.2 ± 0.7 and 0.7 ± 0.3%, respectively). These labelling patterns can be explained by the fact that glutamate is likely synthesized via the citrate synthase reaction from acetyl-CoA and oxaloacetate, with C_1_ coming from the (partially labelled) C_4_ of oxaloacetate, or via the exchange reaction of C1 of 2-oxoglutarate with ^13^CO_2_ that is catalyzed by 2-oxoglutarate synthase. Accordingly, the observed low inorganic carbon fixation is a clear example of heterotrophic CO_2_ fixation.

**Figure 3.**
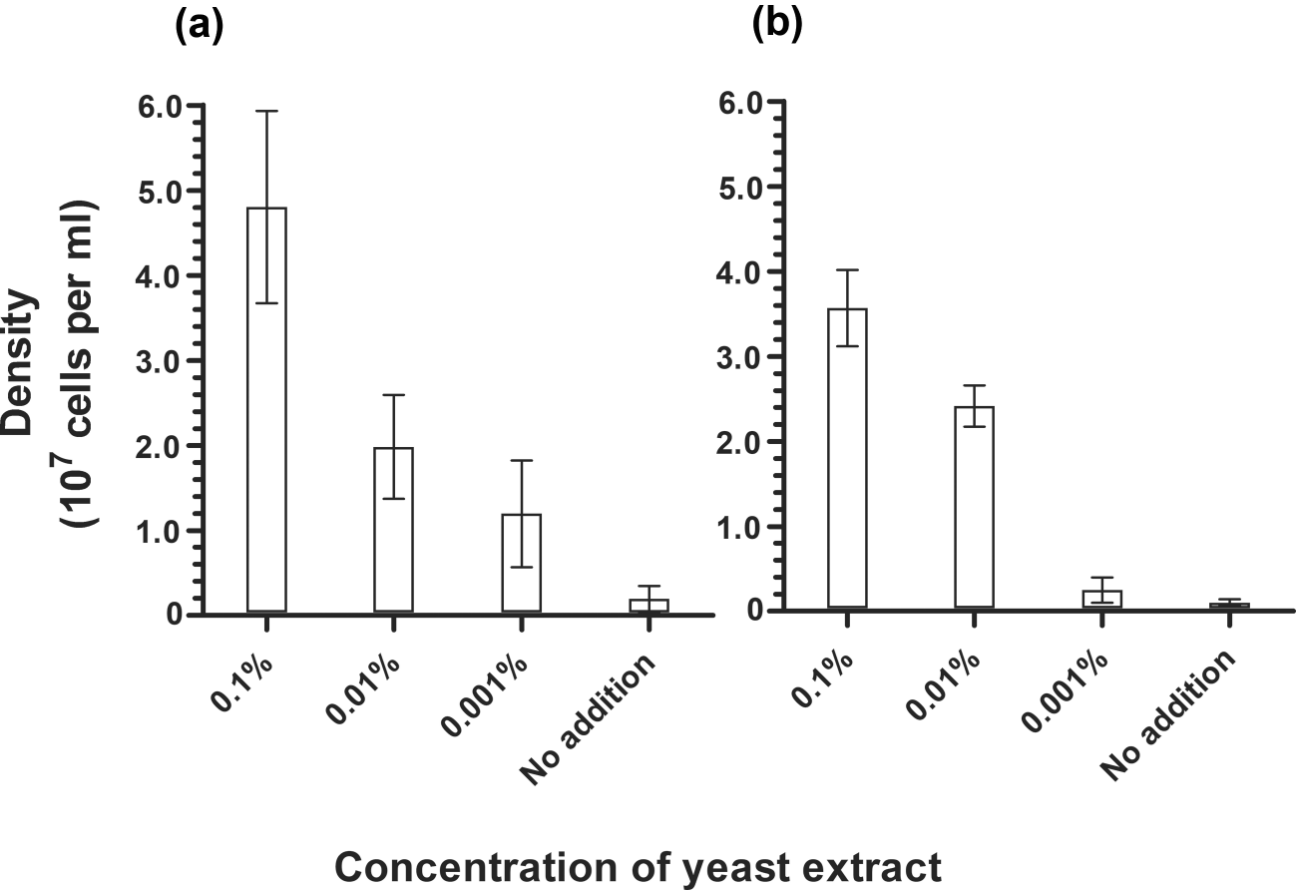
Cell densities of *P. arsenati cum* (a) and *P. aerophilum* (b) cultures supplemented with yeast extract at different final concentrations. Cell densities were measured 3 days after inoculation with a starting cell density of 10^6^ cells/ml. Data are mean ± s.e.m. of at least three biological replicates.

The cultivation of *P. arsenaticum* in the medium that was sterilized with the washed syringe filters was unsuccessful (Fig. 3), highlighting the dependence of the growth of *P. arsenaticum* on the contamination of organic compounds derived from the filters. To conclude, the data obtained in our experiments with *P. arsenaticum* confirm the obligate heterotrophic nature of this archaeon.

### Analysis of *P. aerophilum* autotrophy

For *P. aerophilum*, we did not observe any growth in cultures that were not supplemented with yeast extract (Fig. 3). The lowest possible amount of yeast extract that resulted in a low (10^7^ cells/ml) but sustainable growth was 0.01%. When these cultures were used as an inoculum in a medium without yeast extract, no growth was observed, indicating their purely heterotrophic nature as well.

As the studied *Pyrobaculum* strains were grown from frozen cryostocks prepared immediately after isolation and stored in liquid nitrogen without subsequent re-inoculations, it is unlikely that these species have lost the capability to autotrophic growth due to the constant laboratory cultivation on rich media. Therefore, it appears that both strains are heterotrophs in their environment as well, and their original description as facultative autotrophs probably originated from a misinterpretation of weak growth caused, e.g. by the presence of traces of organic compounds in the medium.

**Figure 4.**
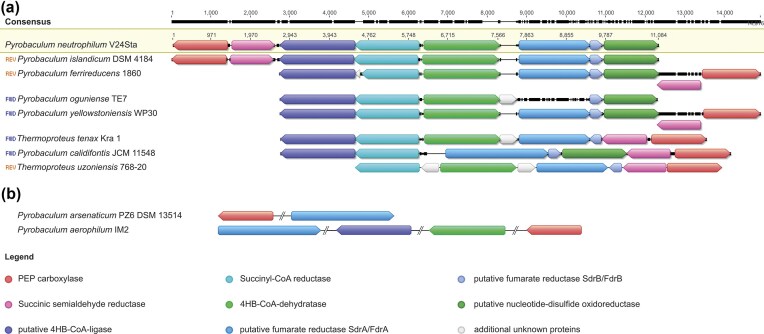
Comparison of the gene cluster encoding enzymes of the DC/HB cycle in sequenced representatives of the *Thermoproteales* order. (a) Alignment of the clustering genes present in the genomes of *P. yellowstonensis, P. ferrireducens, P. islandicum, P. oguniense, P. calidifontis, T. tenax*, and *T. uzoniensis*, using *P. neutrophilum* as a reference. (b) Schematic representation of homologous genes that are also present in the genomes of *P. arsenaticum* and *P. aerophilum*. The genes are colour-coded according to homology, whereas grey genes have no similarity to other genes in the alignment.

Finally, *P. aerophilum* and *P. arsenaticum* are no exceptions among the *Thermoproteales*, as their genomes lack the essential gene sets required for alternative pathways and they consequently do not use an alternative pathway for inorganic carbon fixation. Therefore, the presence of genes encoding key enzymes of the DC/HB cycle appears to be indicative for the capability of *Thermoproteales* to grow autotrophically. We hypothesize that the species described as heterotrophs but possessing the corresponding genes in their genome, may still be capable of autotrophic growth under certain conditions. Our genomic analysis (Table 1, Fig. 4) suggests that *P. calidifontis, P. yellowstonensis*, and *P. ferrireducens*, which were originally described as obligate heterotrophs (Amo et al. 2002, Jay et al. 2015, Slobodkina et al. 2015), may nevertheless be able to grow autotrophically under certain conditions.

In the genome of *P. oguniense*, the gene for PEP carboxylase is located outside the DC/HB cycle gene cluster, while the gene for the succinic semialdehyde reductase is missing (Table 1). Therefore, it is unlikely that this archaeon is able to grow autotrophically, similar to *P. aerophilum*. The *Vulcanisaeta souniana* genome contains homologues of genes for key enzymes of the DC/HB cycle, but these putative homologues are not co-localized and their similarity to the corresponding *P. neutrophilum* genes is lower than for the autotrophic *Thermoproteales* (Table 1). This confirms that *V. souniana* is most likely incapable of autotrophic growth.

In conclusion, one should be wary of particularly weak and unsteady microbial growth, i.e. in the case of cell densities below 10^8^ cells/ml. Such growth can easily be the result of the growth on small amounts of organic compounds derived from the inoculum, present as impurities in chemicals or as contamination from the filtration. The ability of such species to grow autotrophically should be tested in great detail, at best by means of studies that could provide in-depth insight into their metabolism, such as analysis of the ^13^C content of the biomass after growth with ^13^CO_2_ as the gas phase.
